# Biological Performance of Electrospun Polymer Fibres

**DOI:** 10.3390/ma12030363

**Published:** 2019-01-24

**Authors:** Ivan Joseph Hall Barrientos, Graeme R. MacKenzie, Clive G. Wilson, Dimitrios A. Lamprou, Paul Coats

**Affiliations:** 1 Strathclyde Institute of Pharmacy and Biomedical Sciences (SIPBS), University of Strathclyde, Glasgow G4 0RE, UK; ivan.hall-barrientos@strath.ac.uk (I.J.H.B.); graeme.mackenzie@strath.ac.uk (G.R.M.); c.g.wilson@strath.ac.uk (C.G.W.); 2 School of Pharmacy, Queen’s University Belfast, Belfast BT9 7BL, UK

**Keywords:** electrospinning, aligned fibrous scaffolds, cell biology, nanofibers

## Abstract

The evaluation of biological responses to polymeric scaffolds are important, given that the ideal scaffold should be biocompatible, biodegradable, promote cell adhesion and aid cell proliferation. The primary goal of this research was to measure the biological responses of cells against various polymeric and collagen electrospun scaffolds (polycaprolactone (PCL) and polylactic acid (PLA) polymers: PCL–drug, PCL–collagen–drug, PLA–drug and PLA–collagen–drug); cell proliferation was measured with a cell adhesion assay and cell viability using 5-bromo-2′-deoxyuridine (BrdU) and resazurin assays. The results demonstrated that there is a distinct lack of growth of cells against any irgasan (IRG) loaded scaffolds and far greater adhesion of cells against levofloxacin (LEVO) loaded scaffolds. Fourteen-day studies revealed a significant increase in cell growth after a 7-day period. The addition of collagen in the formulations did not promote greater cell adhesion. Cell viability studies revealed the levels of IRG used in scaffolds were toxic to cells, with the concentration used 475 times higher than the EC_50_ value for IRG. It was concluded that the negatively charged carboxylic acid group found in LEVO is attracting positively charged fibronectin, which in turn is attracting the cell to adhere to the adsorbed proteins on the surface of the scaffold. Overall, the biological studies examined in this paper are valuable as preliminary data for potential further studies into more complex aspects of cell behaviour with polymeric scaffolds.

## 1. Introduction

The challenge facing surgeons using mesh scaffolds in tissue engineering applications are two-fold: first, controlling the way the fibres being used physically transform and symbiotically interact with the host body’s physiological function; and more importantly, minimising or negating complications such as chronic infections, development of adhesions, chronic pain and mesh rupture. Drug-loaded oriented scaffolds can be created using a number of processes, including electrospinning, a fibre production method utilising electric force against polymeric solutions. However, despite the various characterisation methods one can perform on these scaffolds, there is a requirement to understand and measure fundamental biological responses. In particular, ideal scaffolds for tissue engineering should be biocompatible, biodegradable, promote cell adhesion and proliferation and have no detrimental effect on cell bioenergetics [1]. Materials such as polycaprolactone (PCL), polylactic acid (PLA) and collagen have achieved biocompatible and biodegradable requirements [2]. However, measuring the effect of any biopolymer on cell adhesion, cell proliferation and cell metabolic activity remains the crucial aspect in determining biocompatibility. Another important biological requirement is assisting the suppression of potential bacterial infections at the site of surgical/scaffold implant. Controlling any bacterial infection is a vital pre-requisite to any potential cell growth, given that the cell/tissue viability in the presence of bacteria such as *Staphylococcus aureus* or *Escherichia* (*E*.) *coli* will be profoundly compromised [3].

*Staphylococcus* (*S.*) *aureus* is a gram-positive bacterium that is commonly found in nasal passages, skin and mucous membranes [4]. This is a major cause of infection of wounds (in particular nosocomial bloodstream infections), especially in surgical procedures that involve medical device implants [5]. Currently, within hernia mesh repair, 68% of infection complications are attributed to the *S. aureus* infections; any infections related to hernia repair will increase the recurrence rates of hernia, meaning that inhibiting the growth of this bacterium will give the patient a better chance of recovery [6]. *E. coli* is another bacterium that can have detrimental effects on the recovery of wounds—it is the most common pathogen found in the hernia sac [7]. This bacterium tends to develop in fluid collections at the site of mesh implant. If this is found at the site of implant, typically drainage of the bacterial fluid and a course of antibiotics are administered (e.g., ceftriaxone and ampicillin) [8]. However, if both *S. aureus* and *E. coli* can be controlled without further administration of antibiotics (which would in turn reduce the possibility of antibiotic resistance), invasive drainage procedures or overall removal of hernia mesh can be avoided – this increases the chance of patient recovery and a better chance of tissue re-growth at a cellular level.

The proliferation of cells related to the healing of wounds is also important within a hernia repair context. Typically, wound healing can be divided into four main steps: (1) haemostasis (0–7 h); (2) inflammation (1–3 days); (3) proliferation (4–21 days); and (4) remodelling (21 days–1 year) [9].

The proliferation period is arguably one of the most important phases given that there is a focus on restoring the tissue network; this can be easily disrupted through any potential infection. Another important aspect of the proliferation stage is the formation of the extracellular matrix (ECM); proper formation of the ECM will help with cell adhesion and regulate growth, movement and differentiation of the cells growing within it. If electrospun scaffolds can mimic the ECM successfully, they may help promote cell adhesion, growth, movement and differentiation [10].

Tissue engineered scaffolds have been used in a number of different clinical applications; in particular, there are a range of applications that are currently being applied within the field of dentistry [11]. Other clinical applications include cardiac tissue engineering (e.g., culturing cells onto a biomaterial scaffold in-vitro and then implanting tissue onto cardiac surface [12]), nerve regeneration for the treatment of stroke (e.g., nanomaterials have been used as a biomimetic in order to induce neuronal growth and guide brain regeneration [13]) and the treatment of pulmonary diseases (e.g., porous scaffolds that mimic alveolar units to allow for greater cell adhesion for lung tissue regeneration [14]).

Studies involving the testing of cellular response against electrospun scaffolds have been have been well reported in the literature: testing cell migration of breast cancer cells (MDA-MB-231) against PCL scaffolds [15], rat periodontal ligament cells against poly(lactic-co-glycolic acid; PLGA) scaffolds [16], human umbilical vein endothelial cell (HUVEC) against PCL–collagen scaffolds [17] and human mesenchymal stems cells against PLA scaffolds [18]. In particular, some of these studies showed evidence that cells typically form confluent monolayers on electrospun scaffolds; fibre orientation affects cell alignment and cells prefer to grow on aligned fibres (e.g., cells showed greater attachment to specifically aligned fibres in comparison to randomly oriented scaffolds) [19]. Though the studies mentioned successfully demonstrated a range of cellular behaviours on electrospun scaffolds, there is a dearth of research that focuses on the cellular response in the presence of drug-loaded electrospun scaffolds.

Therefore, the primary goal of this study was to measure the biological responses of cells against a number of scaffolds (PCL–drug, PCL–collagen–drug, PLA–drug and PLA–collagen–drug variations); cell proliferation was measured with a cell adhesion assay, with subsequent fluorescent and scanning electron microscopy (SEM) imaging and cell viability using resazurin and 5-bromo-2′-deoxyuridine (BrdU) assays. The cell viability assays were chosen specifically (in particular the resazurin assay) for quick determination of cell viability. Other assays, such as the MTT (3-(4,5-Dimethylthiazol-2-Yl)-2,5-Diphenyltetrazolium Bromide) assay, have been used [20]; however, the use of DMSO in this assay has the potential to begin to degrade the polymeric samples. The results in this paper amplify the importance of a number of factors described in previous reports [21,22], such as the importance of hydrophobicity/hydrophilicity of the scaffolds, drug release rates, drug concentrations and fibre morphology.

## 2. Materials and Methods

### 2.1. Materials

PCL with a mean molecular weight of 80 kD, PLA with a mixed molecular weight, irgasan (IRG, variation of Triclosan, >97%), levofloxacin (LEVO, >98%), solvents used for the electrospinning consisting of chloroform (anhydrous, containing amylenes as stabilizers, >99%) and N,N-dimethylformamide (DMF, anhydrous 99.8%) and collagen from calf skin (Bornstein and Traub Type I) were all obtained from Sigma Aldrich (Poole, UK).

### 2.2. Electrospinning of Polymeric Scaffolds

The polymer test specimens were fabricated for each polymeric solution using a custom in-house electrospinning apparatus, which consisted of a syringe pump (Harvard Apparatus PHD 2000 infusion, Cambridge, UK) and two 30 kV high-voltage power supplies (Alpha III series, Brandenburg, UK). The polymer solution was loaded into a glass syringe and fed through tubing with a metal needle tip attached at the end. The needle was clamped into place to allow a high-voltage supply to run through it, which allowed an electric field to be created between the needle and the target plate. The syringe was clamped to a pump, which determined the specific injection flow rate of the polymeric solutions. For each of the three solutions (e.g., unloaded, irgasan-loaded and levofloxacin-loaded), 3 varying flow rates of 0.5, 1 and 1.5 mL·h^−1^ were applied across varying voltages of 2 kV–5 kV (needle) and 10 kV–18 kV (target plate) [21]. The variation in flow rate and applied voltages were to correct any problems that occurred during fabrication, i.e., ‘spitting’ of the solution at the target plate, or any potential beading (which was examined through SEM). The fabrication of this solution was electrospun onto a target that was covered with aluminium foil, in order for the final material to be removed and used for further characterisation. The final yield of electrospun polymers resulted in thin, flexible sheets of material.

### 2.3. Microscopic Characterisation

The morphology and diameter of individual fibres spun from the PCL solution were determined from scanning electron micrographs of each sample (TM-1000^®^, Hitachi, Ltd., Maidenhead, UK). The samples were mounted on an aluminium plate with conductive tape. Images of fibres were taken at various locations of each electrospun PCL scaffold in order to determine the overall uniformity of fibres. Prior to imaging, the samples were sputter coated with gold for 30 s using a Leica EM ACE200^®^ vacuum coater, the process being repeated four times in order to increase the conductivity of the samples. The samples were imaged in secondary electron mode at 5 kV.

### 2.4. Cell Adhesion and Proliferation

Rat aortic smooth muscle cells (RAOSMCs) were explanted from 12-week-old male Sprague-Dawley rats. Aortic rings (5 mm) were placed in T25 flasks in media (10% fetal calf serum (FCS); 50:50 Waymouth and F12; 1% penicillin/streptomycin). The medium was changed every 72 h and growth was monitored using a light microscope until cells reached ~70% confluency (Passage 0). Cells were then split into T75 flasks and allowed to grow to ~70% confluency (Passage 1). Cells were then again split into six well plates, 24 well plates or 96 well plates for further analysis.

Cell adhesion was monitored in 24-well plate dishes with cells seeded directly onto electrospun scaffolds. Square electrospun scaffolds (1 cm × 1 cm) were sterilized using short-wave UV irradiation. Three samples were used per group, plus 1 control (scaffold containing no drug or collagen) and repeated on 2 more well plates. Scaffolds were then pre-wet with media and placed in tissue culture plates. Cells (at the previously mentioned density) in a volume of 100 μL were seeded directly onto each scaffold, incubated at 37 °C for 4 h in order for the cells to adhere to the surface. Each well was then filled with 2 mL of growth medium.

Initially, the RAOSMCs were left for 3 days to judge the adhesion of cells on the scaffold surface, and then RAOSMCs were tested for proliferation within 14 days of seeding. In this case, media was replaced every 3 days, and scaffolds were removed at 3, 7 and 14 days in order to image cells on the scaffold (using fluorescent microscopy). Adhesion and proliferation were determined through the staining of dead cells using propidium iodide (PI), a red-fluorescent nuclear and chromosome counterstain. This process involved lifting the scaffolds from the wells, washing them with PBS buffer and fixing the cells using 5% paraformaldehyde. Samples were then placed in 1 mL of PBS buffer, and 10 μL of PI solution was added and left in dark storage for approximately 30 min. Samples were then placed on a coverslip, then subsequently imaged on a Nikon Eclipse E600 Epiflurorescent Upright Microscope (Nikon, New York, NY, USA), at wavelengths of 495 nm (FITC) and 532 nm (TRITC). Cells were then counted using ImageJ software (Version 1.52, National Institutes of Health, Wisconsin, WI, USA). Images were converted to greyscale; the image threshold was altered to display a black and white image highlighting the cell nuclei. A watershed function was then applied to separate cells that were grouped close together, and a particle analyser was run at a measurement of minimum 100–120 pixels and a circularity range of 0.00–1.00.

### 2.5. Cell Viability Assay

In order to measure the cell viability against the levels of drug used in the electrospun scaffolds, a resazurin assay was performed. Cells (RAOSMCs) were grown in 1% FCS, 10% FCS, 1% addition of IRG or addition of 0.5% addition of LEVO, at a density of 3 × 10^4^ cells/cm^2^ (in 24-well plates) for 3 days. The resazurin reagent was then added to each well, incubated at 37 °C for 4 h, then subsequently tested for fluorescence using a fluorometer at 560Ex nm and 590Em nm.

Ranges of drug concentrations (IRG = 1%, 0.1%, 0.01%, LEVO = 1%, 0.5%, 0.1%) were then tested against the growth of cells (RAOSMCs).

### 2.6. Cell Proliferation Assay

A 5-bromo-2′-deoxyuridine (BrdU) assay was used to test the proliferation of cells (Sigma Aldrich, Poole, UK). Cells were grown at a density of 3.6 × 10^4^ cells/100 μL (in 96-well plates) for 3 days. Cell proliferation was assessed based on 5-bromo-2′-deoxyuridine (BrdU) incorporation followed by ELISA according to the manufacturer’s protocol. BrdU solutions were added to the well to achieve 1 nM final concentration and incubated for 6 h. Cells were then washed, fixed and denatured at 37 °C for 30 min. Following successive washings with PBS, cellular BrdU uptake was detected by colorimetric ELISA (450 nm) using anti-BrdU antibody. Data were normalized to the protein concentration of the corresponding sample.

### 2.7. Statistical Analysis

All experiments were performed in triplicate with calculation of means and standard deviations. Two-way analysis of variance (ANOVA) was used for multiple comparisons along with Tukey’s multiple comparing tests, followed by a T-test to access statistical significance for paired comparisons. Significance was acknowledged for *p* values lower than 0.05.

## 3. Results

### 3.1. Cell Adhesion and Proliferation

**Figure 1:**
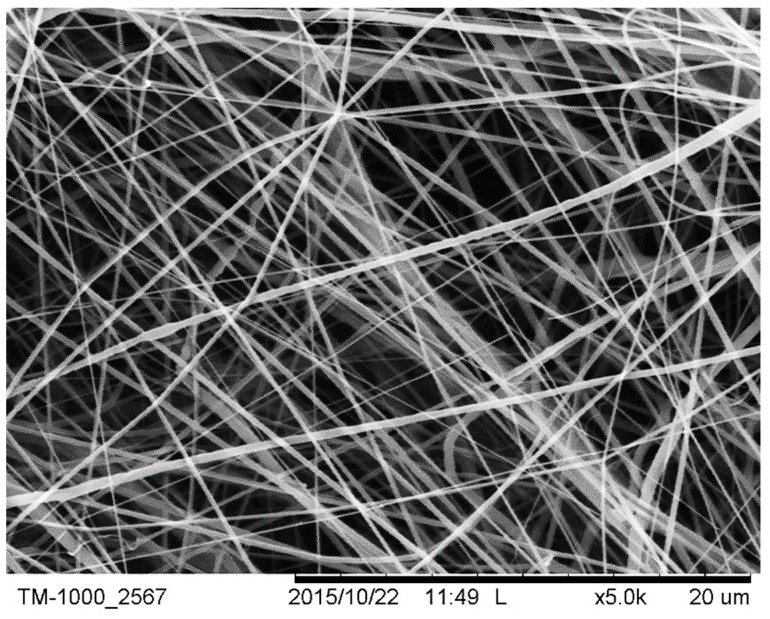
SEM image of polycaprolactone (PCL)–Collagen–levofloxacin (LEVO) fibres at 20 µm scale.

**Figure 2:**
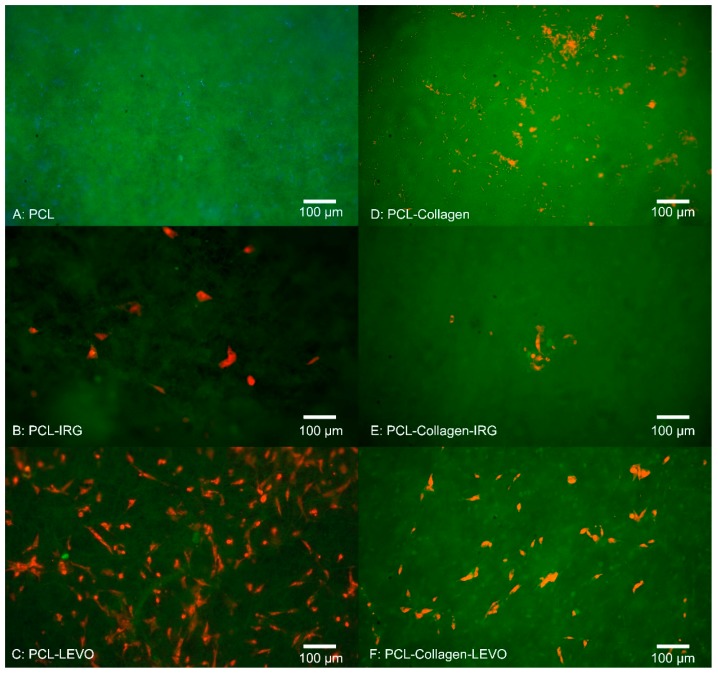
Fluorescent images of cell adhesion assay for rat aortic smooth muscle cells (RAOSMCs) after 3 days (**A**) PCL (**B**) PCL–irgasan (IRG) (**C**) PCL–LEVO (**D**) PCL–collagen (**E**) PCL–collagen–IRG (**F**) PCL–collagen–LEVO.

**Figure 3:**
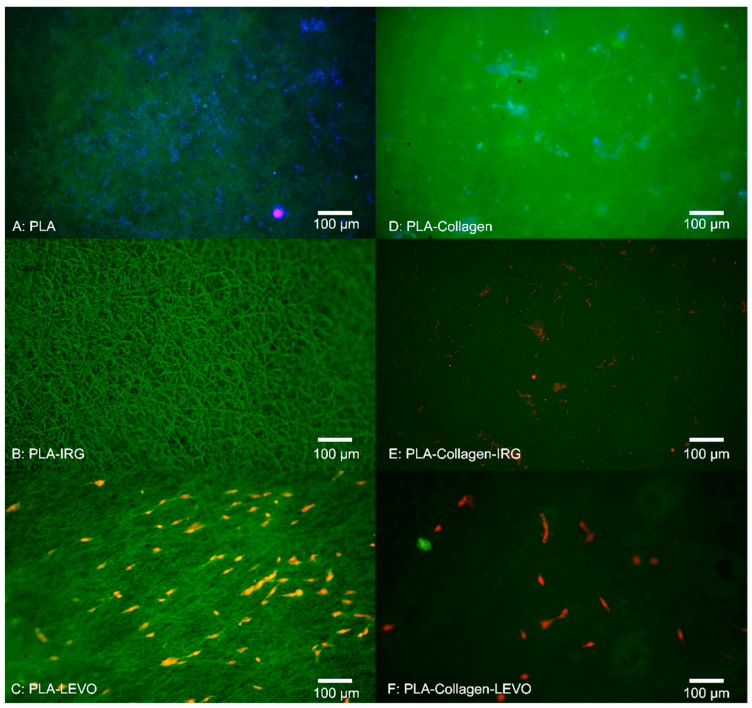
Fluorescent images of cell adhesion assay for RAOSMCs after 3 days (**A**) polylactic acid (PLA) (**B**) PLA–IRG (**C**) PLA–LEVO (**D**) PLA–collagen (**E**) PLA–collagen–IRG (**F**) PLA–collagen–LEVO.

The uniformity of fibres is shown through an SEM image in Figure 1. Initial 3-day studies of RAOSMC growth against all PCL and PLA variations can be seen in Figure 2 and Figure 3. The fluorescent images in Figure 2 indicate there was no adhesion of cells on the PCL or PCL–collagen scaffolds, and there appeared to be presence of a bacterial infection (bacteria not identified).

RAOSMC adhesion was found to be minimal within both PCL–IRG and PCL–collagen–IRG samples, with a cell density of approximately 1600 cells/cm^2^ and 500 cells/cm^2^. PCL–LEVO, however, showed a cell adhesion density of approximately 14,000 cells/cm^2^ and 7500 cells/cm^2^ for PCL–collagen–LEVO.

Figure 3 indicates that there was no apparent cell adhesion of RAOSMCs against the PLA, PLA–collagen and PLA–collagen–IRG scaffolds, with only a bacterial infection appearing in each case. There was no cell adhesion measured for the PLA–IRG scaffold; however, no bacterial infection was observed. Cell adhesion was observed for PLA–LEVO and PLA–collagen–LEVO scaffolds, with a cell count of approximately 11,000 cells/cm^2^ and 2000 cells/cm^2^, respectively.

**Figure 4:**
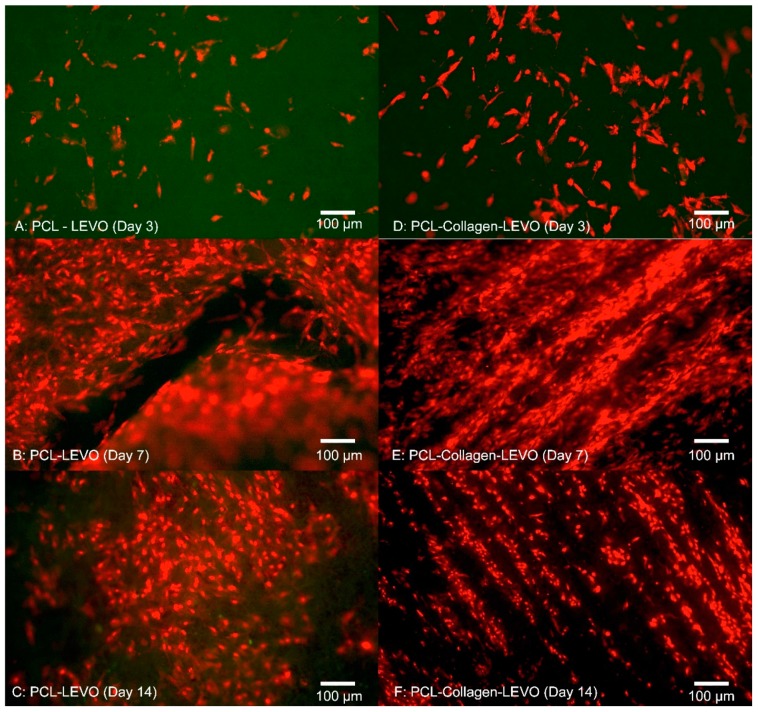
Fluorescent images of cell proliferation of RAOSMCs (**A**) PCL–LEVO after 3 days (**B**) PCL–LEVO after 7 days (**C**) PCL–LEVO after 14 days (**D**) PCL–collagen–LEVO after 3 days (**E**) PCL–collagen–LEVO after 7 days (**F**) PCL–collagen–LEVO after 14 days.

**Figure 5:**
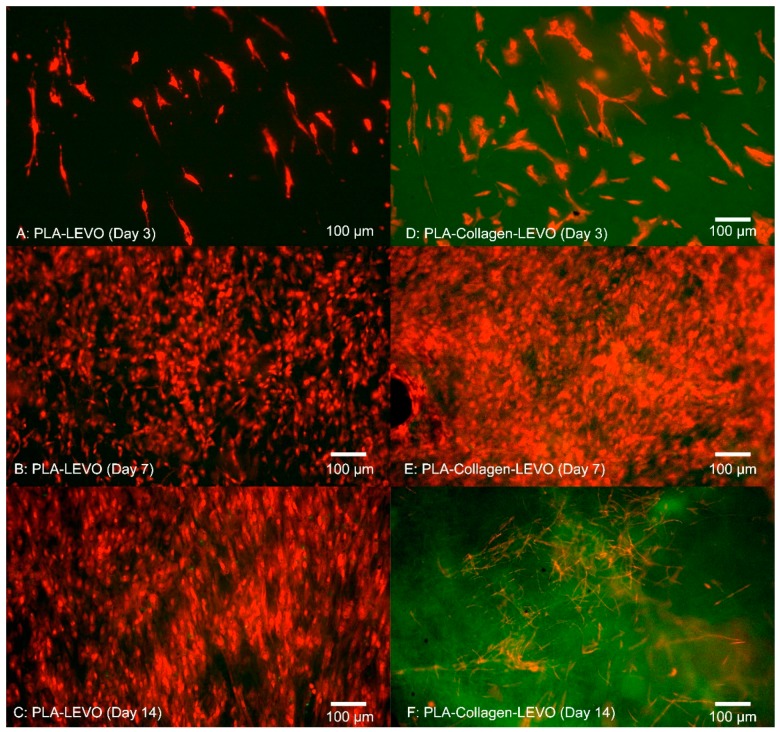
Fluorescent images of cell proliferation of RAOSMCs (**A**) PLA–LEVO after 3 days (**B**) PLA–LEVO after 7 days (**C**) PLA–LEVO after 14 days (**D**) PLA–collagen–LEVO after 3 days (**E**) PLA–collagen–LEVO after 7 days (**F**) PLA–collagen–LEVO after 14 days.

A 14-day study was conducted on PCL–LEVO and PLA–LEVO combinations due to the highest measurement of proliferative cells in the previous 3-day study. The results from this 14-day study can be seen in Figure 4 and Figure 5. PCL–LEVO showed a cell count across 3, 7 and 14 days of 6800, 140,000 and 98,000 cells/cm^2^. PCL–collagen–LEVO scaffold had a cell count across 3, 7 and 14 days of 1500, 180,000 and 130,000 cells/cm^2^.

Similar to the results of the 3-day study with PCL–LEVO/PCL–collagen–LEVO, greater proliferation was measured against the PLA–LEVO and PLA–collagen–LEVO scaffolds; therefore, the 14-day study was tested against the scaffolds. PLA–LEVO scaffolds showed an average cell count of 7800, 128,000 and 119,000 cells/cm^2^ at 3, 7 and 14 days. PLA–collagen–LEVO scaffolds showed an average cell count of 12,800 and 168,000 after 3 and 7 days, respectively, and at 14 days was devoid of cells (a bacterial infection was observed).

**Figure 6:**
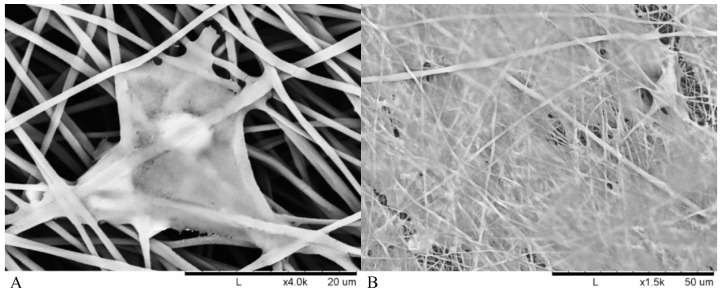
SEM images of adhered RAOSMC in polymer fibres (**A**) 40 µm image at 3 days (**B**) 120 µm image at 14 days.

An SEM image was also taken (Figure 6) that shows how the cells initially adhered within the fibres after 3 days with subsequent proliferation after 14 days.

### 3.2. Cell Viability

**Figure 7:**
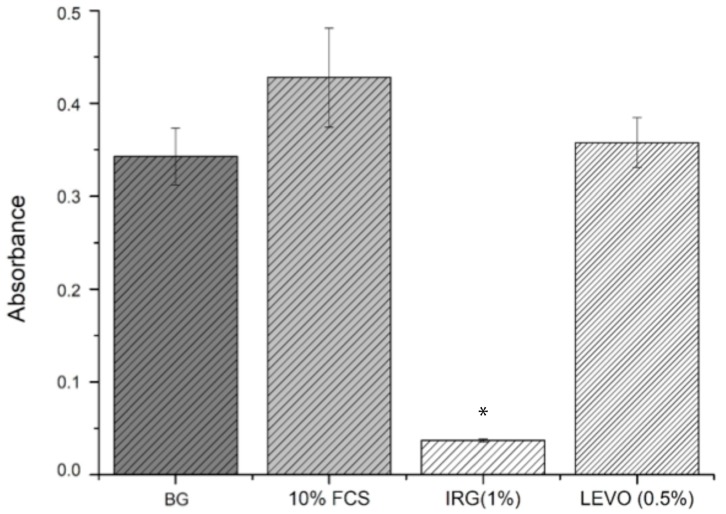
Absorbance data with standard error bars (n = 3) for resazurin assay against BG (background) 10% fetal calf serum (FCS), 1% IRG and 0.5% LEVO concentrations. BG, IRG (1%), LEVO (0.5) vs. 10% FCS * *p* < 0.05.

Data relating to the absorbance of resorufin from the resazurin assay can be seen in Figure 7. Following the addition of 10% FCS to cells, metabolic activity increased by 24.82 ± 9.2% when compared with quiesced cells in 0.1% FCS. The effect of 1% IRG had a detrimental effect on metabolic activity, reducing it by 89.23 ± 0.48% below background activity (0.1% FCS). The effect of LEVO was less harsh when compared with IRG, with an observed increase of 4.35 ± 7.89%. IRG reduced activity by 91.37 ± 0.61% against 10% FCS. LEVO reduced metabolic activity against 10% FCS by 16.39 ± 5.41%.

**Figure 8:**
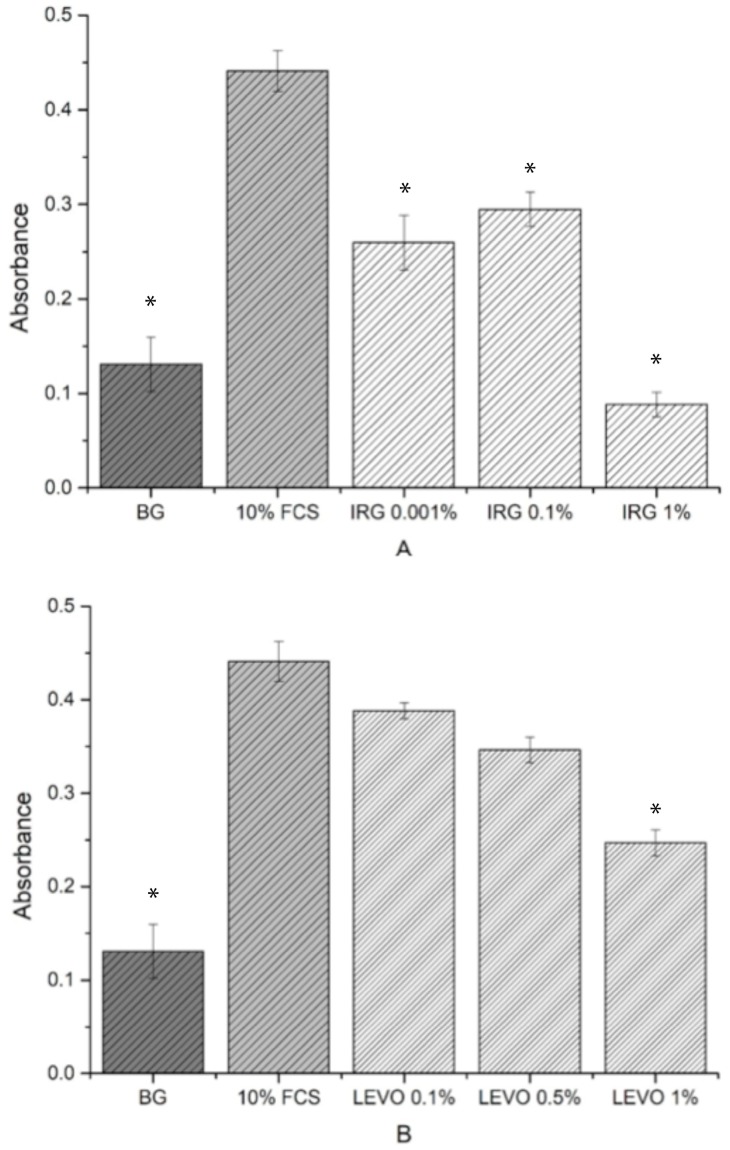
Absorbance data with standard error bars (n = 3) for 5-bromo-2′-deoxyuridine (BrdU) assay against (**A**) background, 10% FCS, 0.01% IRG, 0.1% IRG, 1% IRG. BG, IRG (0.001%), IRG (0.1%), IRG (1%) vs. 10% FCS * *p* < 0.05. (**B**) background, 10% FCS, 0.1% LEVO, 0.5% LEVO and 1% LEVO concentrations. BG, LEVO (0.1%), LEVO (0.5%), LEVO (1%) vs. 10% FCS * *p* < 0.05.

BrdU data is shown in Figure 8A,B. In comparison with the background, a high cell proliferation was observed with the addition of 10% FCS. For the range of IRG concentrations, cell proliferation increased for 0.01% and 0.1% concentrations and decreased in proliferation for 1% concentrations. LEVO concentrations generally showed a higher proliferation compared with the background, with cell proliferation reducing as the concentration increased from 0.1% to 1%.

## 4. Discussion

Understanding the behaviour of smooth muscle cell (SMC) growth against scaffolds is important, given the importance of SMCs within the development of tissue re-growth. Initial 3-day studies of RAOSMCs against a number of PLA scaffold combinations proved insightful in determining which scaffold/drug exhibited greatest efficacy in allowing cells to adhere. Bacterial infections were only observed in the scaffolds that did not contain any antibacterial drug embedded within the polymeric fibres. The source of the bacterial contamination was likely due to the tissue isolation procedure from the animals. Of course, any infection within the culture model is undesirable. However, in this case what is noteworthy is contamination was prevented in all cases by the antibiotic combinations. With respect to translational relevance, this is a potential verification of the potential value of this antibiotic/polymer combination as a candidate for use in a surgical/clinical scenario of mesh implant procedure where infection is highly possible. Typically, bacterial cells have been shown to invade endothelial and smooth muscle cells in particular [23]. The addition of IRG across both PCL and PLA samples proved detrimental to RAOSMCs adhesion. This may be largely due to the high hydrophobicity of IRG in combination with two types of polymers that are generally hydrophobic in nature—hydrophobicity typically hinders cell adhesion and proliferation [24].

There appeared to be a greater adherence of cells on LEVO-loaded scaffolds that did not contain type I collagen; in the case of the PCL–LEVO and PCL–collagen–LEVO samples, both have the same hydrophilic characteristics. However, the difference in cell adhesions cannot be attributed to this. It is more likely the cells were interacting as a consequence of the variance in fibre diameters for the two scaffolds, or the release of levofloxacin inhibiting bacteria may have affected cell adhesion. Concerning the cell attachment against fibre diameter, it should be the case that scaffolds (in particular PCL scaffolds) with smaller diameters (such as PCL–collagen–LEVO, with an average fibre diameter of 1.5 µm) would have better attachment of cells due to the increase in specific surface area [25]. However, this was not the case. Thus, the decrease in cell attachment for the PCL–collagen–LEVO scaffold may be due to factors that include drug release rate (there is a higher release percentage of drug from the PCL–LEVO scaffolds compared with the PCL–collagen–LEVO scaffold (as demonstrated in previous studies [22]), and an increase in bacterial resistance may help with cell adhesion), or simply that the number of cells seeded onto the scaffolds were lower. PLA–LEVO and PLA–collagen–LEVO scaffolds showed a lower cell adhesion count. This lower cell adhesion may have been attributed to the high hydrophobicity (water contact angle ~108° for PLA–LEVO and ~132° water contact angle for PLA–collagen–LEVO; these data sets were published in previous works [21]) and smaller fibre diameter for the PLA–collagen–LEVO. Although a smaller fibre diameter could encourage cell attachment, having fibres that range between 100 and 300 nm (of which there are some measured in the PLA–collagen–LEVO scaffold) will not have a significant impact in the promotion of cell adhesion [17].

One explanation that the RAOSMCs are adhering and proliferating across the LEVO-loaded scaffolds (for both PCL and PLA) may be due to the varying surface charges. It has been shown in several studies that the most suitable conditions for cell adhesion and proliferation occur when there is presence of negative ions associated with the surface treatment or drug of interest [26]. In particular, a carboxylic (–COOH) functional group is found predominately in the levofloxacin compound; this functional group has a negative charge, with hydrophilic properties, and interacts almost exclusively with fibronectin [27]. This interaction with fibronectin (which is typically found within the extra cellular matrix of cultured cells) is important, because if the fibronectin protein is adsorbed onto the scaffold surface, this allows for the anchoring of fibronectin to the cell [28]—in this case allowing the RAOSMC to adhere to the scaffold surface.

Cell adhesion within the fibrous scaffolds can be seen in Figure 6. The structure of the cell took shape according to the fibrous network it was surrounded by, extending in multiple directions according to whatever direction a fibre was oriented. This was due to cells and nuclei becoming elongated in order to adapt to the fibre structure, with the cell contracting in order to accommodate within the fibrous network of the electrospun scaffolds [29]. A high confluence of cell proliferation can be seen in Figure 6B, suggesting that initial success in cell adhesion allowed the smooth muscle to proliferate over 14 days. 

Examining the cell proliferation of RAOSMCs over 14 days was useful in demonstrating the efficacy of the scaffolds against cell growth. We have demonstrated that cells had a higher level of proliferation and confluence at 7 days (across PCL and PLA scaffolds), followed by a decrease in proliferation at 14 days for most scaffolds (Figure 3 and Figure 4). This decrease may be explained due to the inhibition that is typically experienced when cell proliferation is at a high confluence [30]. This particular cell line (RAOSMCs) also stops growing, and cells enter a senescence phase upon reaching confluency [31]. Despite a reduced number of cell adhesions observed in scaffolds containing collagen, this did not affect cell proliferation from 7 to 14 days. There is a possibility of the hydrophobic bands (from collagen fibrils) being drawn to the surface of the fibres; therefore, it may be the case that any cell–collagen interactions occurring may have been delayed due to hydrophobic forces [21].

We also observed that smooth muscle cells were growing in ‘lines’ on the PCL–collagen–LEVO scaffold. This may be due to the fibre orientation of the sample, as fibres can be oriented in one direction simply by stretching the scaffold in one direction. It may be the case that the samples were stretched during the setup process (samples had to be removed from their aluminium backings, with PCL–collagen–LEVO demonstrating high adhesion to the aluminium). However, this is an important factor as cells will typically elongate and grow along a particular direction of aligned fibres [32]. 

Cell viability studies were useful in determining whether the levels of drug loaded into the scaffolds had any potential toxic or inhibitory effects on the growth of RAOSMCs. It was clear from the results that the concentration of IRG used (1%) was detrimental to cell growth and viability. IRG has an EC_50_ value of approximately 0.0012 mg/mL [33]. Given that the 1% concentration used in the resazurin assay was 0.57 mg/mL (475 times higher than the EC_50_ value), the reason for poor cell adhesion and proliferation on IRG-loaded scaffolds was due to this high concentration. The BrdU assays were useful in demonstrating the varying IRG concentrations directly against the proliferation of smooth muscle cells—with 0.1% and 0.01% concentrations showing a more favourable response compared to the 1% IRG. It should also be noted that IRG has a mode of action where the drug can break through membranes and demonstrate inhibitory effects, such as blocking of lipid biosynthesis [34], which may be another plausible explanation as to why there was poor cell adhesion and proliferation.

We observed that the concentration of LEVO (0.5%) used in the initial resazurin study had no measurable effect on cell metabolic activity when compared with controls. At this stage, it would be safe to judge that the concentration of LEVO used was not detrimental to the growth and proliferation of RAOSMCs. Further evidence of this was shown in the BrdU results, indicating that an increase in concentration (to 1%) of LEVO decreased the proliferation of cells and that a decrease in concentration (to 0.1%) permitted cell proliferation. This may be linked to the quoted EC_50_ value of 0.0074 mg/mL [35]—0.1% of LEVO used was 0.024 mg/mL, which is closer to the EC_90_ value.

## 5. Conclusions

Examining the cellular behaviour against polymeric scaffolds has proven extremely useful in determining whether they have potential use for further development within the tissue-engineering field. One of the main goals set out in this research was concerning how smooth muscle cells proliferate/are compatible against the various scaffolds, and a number of important outcomes can be taken from the results and discussion. Firstly, the use of irgasan within the polymeric scaffolds proved to be detrimental to the adhesion and proliferation of cells. This was due to the concentration of IRG used being far greater than the EC_50_ value quoted in various publications, which meant that it was toxic to the cells growing in culture. Secondly, levofloxacin-loaded scaffolds showed the greatest number of cell adhesions and subsequent proliferation over 14 days. This could be explained by the hydrophilic nature of the drug (cells have a preference to grow in hydrophilic conditions); however, a very high hydrophobicity of the PLA–collagen–LEVO scaffold was reported in our previous studies. A more plausible explanation may be that the negatively carboxylic acid group found in LEVO was attracting positively charged fibronectins, which in turn were attracting the cell to adhere to the adsorbed proteins on the surface of the scaffold. Overall, the biological studies examined are useful as preliminary data for potential further studies into more complex aspects of cell behaviour: for example, understanding the behaviour of fibroblasts against electrospun scaffolds will help in understanding potential scar formation at the site of insertion (e.g., by reducing scar tissue formation will help prevent mesh adhesion) and in measuring inflammatory factors such as transforming growth factor beta 1 (TGF-β1) and interleukin 6 (IL-6).

## 6. Study Perspective and Future Directions

This present study forms the first part of a planned program of work that may lead to the first human clinical trials of a bio-compatible novel drug eluting polymer for mesh implant surgery. In this study we have identified a number of important observations using in-vitro cell studies combined with key drugs–polymer combinations. Our next stage will progress and develop this work to in-vivo studies where the focus will be tissue integration and a systems biology assessment of our drug eluting polymer mesh implants.
